# Long-lived compositional heterogeneities in magma chambers, and implications for volcanic hazard

**DOI:** 10.1038/s41598-019-40160-1

**Published:** 2019-03-01

**Authors:** Deepak Garg, Paolo Papale, Simone Colucci, Antonella Longo

**Affiliations:** Istituto Nazionale di Geofisica e Vulcanologia, Sezione di Pisa, Via della Faggiola 32, 56126 Pisa, Italy

## Abstract

Magmas discharged during individual volcanic eruptions commonly display compositional variations interpreted as new arrivals at shallow depth of more primitive, hotter, volatile-rich magma batches mixing with resident, colder, partially degassed magma. Heterogeneities in eruption products are often interpreted as evidence of short times of order tens of hours from new magma arrival to eruption, raising concerns for emergency planning. We show here, through numerical simulations, that magma convection and mixing in a shallow magma chamber can result in long-lived, dynamically stable configurations with coexistence of magmas from nearly pure to variably mixed end-member compositions. Short mixing time scales may therefore relate to sin-eruptive processes, as heterogeneities found in the eruptive products are not necessarily the fingerprint of new magma arrival shortly preceding or triggering the eruption.

## Introduction

Mixing of magmas with different temperature and/or bulk composition including different viscosities and volatile contents is an important petrogenetic process for both igneous petrology^1^ and volcano dynamics^2^. Typically, a low-density, volatile-rich, primitive, hot magma ascends in the crust and interacts with previously stored, more chemically evolved, partially degassed and denser magma^1^. The degree of magma mixing, which spans a continuum from mechanical mingling to complete homogenisation, depends upon the magma properties, the driving forces, and the time available for mixing.

Evidence of mixing and compositional heterogeneities are almost ubiquitous in the arc-volcanism. Heterogeneous andesite-dacite ejecta in the pyroclastic deposits of Tongariro volcano have been interpreted^3^ as result of multiple magma-mixing-mingling events. The same authors found similarities with the products from 1995 eruptions of the neighbouring Ruapehu volcano^4^. Evidence of mixing of andesitic and dacitic magma during the Mount Rainier eruption^5^ comes from macroscopically banded pumice, as well as phenocryst, matrix glass, melt inclusion, and whole rock compositions. The authors find that the time involved from the mixing event through the eruption is limited to a period of 4–5 days based on Fe-Ti oxide re-equilibration. The Mount Rainier eruption became more dacitic over time, and the final products show evidence of partial re-equilibration between the andesite and dacite. At Mount St. Helens, a prominent role of magma mixing is suggested by strong physical evidence, such as banded pumices, thermal heterogeneities in single pumices, phenocryst disequilibrium, contrasts between compositions of glass inclusions and host matrix glass, and amphibole reaction rims^6^.

The physics of magma mixing has been studied through numerical simulations^7–17^ and experiments with both synthetic and natural compositions^18–26^. In^8^, the authors simulate, in a 2D rectangular box, the mixing dynamics in melt-dominated plutonic rocks by considering two magma bodies separated by a vertical interface. The authors solve the transport equations adopting a modified Boussinesq approximation and neglecting the inertial terms. For an andesite-dacite system they obtain mixing time scales in the order of years. In^9^, the above model was extended to a multiphase mixture of silicate melts and crystals, but neglecting buoyancy associated with temperature change. In^10^, the authors simulate same kind of crystal-bearing melt with different gas bubble content, in a rectangular box, using an isothermal multiphase numerical model, obtaining a time scale of order one hour. In^12,13,16^, the authors simulate, using a 2-D FEM code, the mixing dynamics in a melt-dominated, multipe chamber + dyke system, as due to buoyancy-driven shallow chamber replenishment. In^27^ the authors, on the base of simple scaling relatioships, estimate the ascent speed for basalt-andesite magma in a chamber to be as low as 5 to 20 m/day, which results in mixing time scales of several decades. However, for the same composition, other authors^28^ estimate mixing time scales ranging from 1.4 h to 12 days by studying the reaction coronas around quartz crystals and the dissolution textures of plagioclase. More recently, the time scale of magma mixing has been evaluated through forced mixing experiments at high-temperature and atmospheric pressure in a centrifuge furnace^26^, showing that complete homogenisation of the system occurs within a few hours, reducing to a few minutes if mechanical mixing results in filaments of compositionally different magmas having mm-scale thickness^29^. Mixing time scales of tens of minutes, interpreted as the time available for mixing from new magma arrival into the magma chamber to eruption, have been described for three eruptions at Campi Flegrei based on diffusion profiles and comparison with experiments^26^. Such short time scales raise serious concerns for emergency preparedness purposes. In fact, if a new episode of magma rise can lead to an eruption in tens of minutes or in a few hours, then the time since when such an event can be detected from volcano monitoring, to the occurrence of the eruption, can be far too short to allow any short-term risk mitigation measure, such as evacuation of inhabited areas.

The above shows that the time evolution of magma mixing, and the rate at which hybrid compositions are generated, is still largely a matter of debate. The current views reflect into a range of interpretations and hypotheses, in particular on the time span between new magma injection and eruption, leading to diverse scenarios with substantial implications for volcanic hazards. Therefore, additional investigation is needed both for fundamental understanding of processes and dynamics, and for volcano emergency planning and operations. Here we study the thermo-fluid dynamics of mixing, in a sill-like geometry, between pure melt andesite and dacite magmas having distinct temperature, composition, viscosities and volatile abundance, using a 2-D FEM code^12,30^. We refer to mixing in a macroscopic sense, as we resolve the process at 1 m scale, whereas chemical mixing occurs at the scales of molecules.

Five numerical simulations of an andesite-dacite system are performed by varying viscosity and volatile contents (Table 1). In order to cover a spectrum of possible conditions and investigate the roles of some relevant quantities involved in the convection and mixing processes, we perform a parametric study by varying the volatile contents of the two magmas (and accordingly, their computed properties), and by multiplying the computed viscosities of both magmas by a constant factor. The employed model is discussed with further details in the Methods section, as well as in previous work^12,13,16,31^ of us. Here it is useful to remark that i) the transport equations fully account for magma compressibility, inertial, viscous and gravity forces, temperature differences and exchange between the two magmas, and phase change between the gas and melt phase; and ii) the relevant properties density, viscosity and heat capacity, as well as multi-component (water and carbon dioxide) gas-melt equilibrium, are computed locally in space and time as a function of the local physical and chemical conditions as they evolve during magma convection and mixing. Although the model includes complex, non-Newtonian rheology, analysis of the conditions and of the numerical results show that within the range of our numerical simulations, non-Newtonian effects are negligible. Therefore, we have limited the calculations to locally (space-time) defined, temperature and composition (melt oxides and gas bubble concentration)-dependent Newtonian rheology.

**Table 1:** Composition of magmas and heat capacity of oxides, and list of simulations.

	SiO_2_	TiO_2_	Al_2_O_3_	Fe_2_O_3_	FeO	MnO	MgO	CaO	Na_2_O	K_2_O
Andesite	58.70	0.88	17.24	3.31	4.09	0.14	3.37	6.88	3.53	1.64
Dacite	65.98	0.59	16.15	2.47	2.33	0.09	1.81	4.38	3.85	2.20
*c*_*p*_(*Jkg*^−1^*K*^−1^)	1331	1392	1545	1434	1100	—	2424	1781	1651	1030
**Simulation**	**Andesite**		**Dacite**				**Viscosity**		**Simulated time**	
	**H**_**2**_ **O wt%**	**CO**_**2**_ **wt%**	**H**_**2**_ **O wt%**		**CO**_**2**_ **wt%**		**(10** ^**3**^ **Pa.s)**		**(h)**	
1	4	2	4		0.1		6.2–32.0		1.45	
2	4	2	4		0.1		18.8–96.2		1.30	
3	4	2	4		0.1		62.9–320.8		2.45	
4	2	1	2		0.1		12.2–123.1		1.15	
5	6	3	5		1		5.7–44.4		1.15	

**Figure 1:**
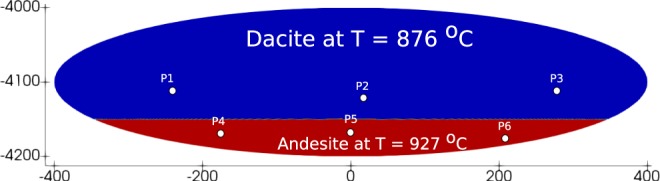
Magma chamber domain and initial setup. P1-P6 are Lagrangian tracing points (see Fig. 7e,f below).

The simulation conditions have been selected to be within typical ranges for andesitic volcanism. Total water and carbon dioxide contents (dissolved in the melt plus exsolved in the gas phase) vary in the range 2–6 and 0.1–3 wt%, respectively. The magma chamber domain is modelled as a commonly observed sill-like shape^32,33^. The top of the oblate-elliptical chamber is at 4 km depth and the major and minor axes are, respectively, 800 and 200 m long. Within the frame of the 2D Cartesian simulation approach employed here, and considering a third dimension significantly longer than the two simulated ones, the volume of the sill-like chamber turns out to be a few tenths of km^3^, within the range of shallow reservoirs below andesitic volcanoes (e.g., Mount Rainier^5^, St. Helens^34^). In the simulations, andesite takes up the bottom part of the chamber at a temperature of 927 °C, while the upper part hosts dacite at a temperature of 876 °C. A horizontal interface, at 4150 m depth, separates the two magmas (Fig. 1). The initial pressure distribution is computed considering the lithostatic load at the chamber roof and a horizontally uniform magmastatic profile. Most estimates of magma chamber conditions indicate^35^ very small Biot number ≪1, i.e., the heat transfer is limited by the thermal resistance of the wall rock. Therefore, the chamber walls are set adiabatic (zero heat flux) with zero velocities (no-slip condition).

Current interpretations of magmatic systems^36^ tend to portrait them as being made of crystal mushes at deep crustal levels, evolving to melt-dominated magmas at more shallow levels. Such a view is generally consistent with our simulation set up (shallow depth, melt-dominated magmas). We emphasize, however, that no systematic observations of magma chamber conditions exist. The serendipitous encounter with rhyolitic magma during geothermal well drilling at Krafla caldera, Iceland, in 1984^37^ unexpectedly revealed only samples of either crystallized felsite or nearly aphyric magma^38^, questioning the commonly accepted view of crystal-rich mush constituting the walls of a magma chamber.

It is worth discussing here that the processes occurring in a magmatic system are such that an initial condition as in Fig. 1 may be difficult to be realized in nature. More likely, as long as batches of lighter magma reach the chamber, they will be displaced upwards by buoyancy forces, hindering the possibility of buoyant magma accumulation at chamber bottom^16^. However, conditions characterized by an initial unstable interface as in Fig. 1 have been largely employed to study convection in magma chambers^8–10^. Initial compositional interfaces are also employed to study magma mixing in forced convection experiments^26^. We anticipate that more consistent initial conditions require extending the simulations to larger domains including the deep portions of a magmatic system from where volatile-rich magmas migrate towards shallow levels (e.g., as in^12,16^). At the same time, we believe that a simple configuration like in Fig. 1 embeds many aspects of the convection/mixing processes and compositional evolution in a shallow magma chamber, besides mimicking the initial sharp interface set in mixing experiments. Focusing on magma chamber only has the additional advantage of allowing better spatial resolution while keeping the computational efforts affordable.

We show that in the first few minutes after disruption of the unstable magma interface, efficient mixing dominated by buoyancy forces occurs, while at later times convection layers form and mixing becomes progressively less efficient due to decrease of temperature and compositional gradients. Over the time-scale of our simulations (a few hours) the efficiency of mixing is largely controlled by the viscosity of magma and volatile contents, with low viscosity favouring the attainment of higher degrees of homogenisation. However, complete homogenisation requires much longer time well beyond our simulated time scale. We demonstrate that magma mixing processes can generate a long lived, highly heterogeneous magma, in some cases preserving the end member compositions.

## Results

**Figure 2:**
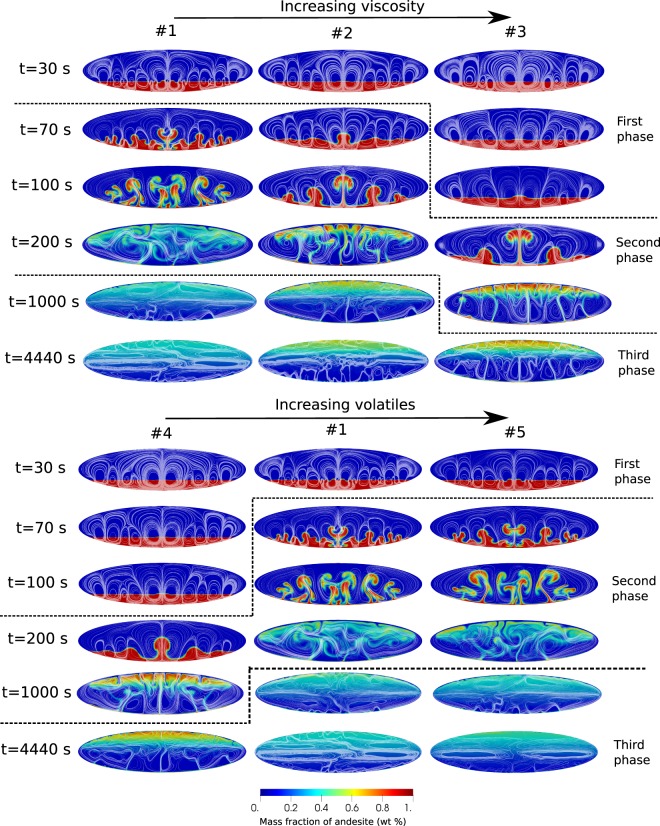
Magma mixing results for increasing viscosity and increasing volatile content (see Table 1). Streamlines of velocity are superimposed to composition.

Figure (2) shows the temporal evolution of the mass fraction of andesite in the magma mixture for increasing viscosity and volatiles. Three different phases can be distinguished in the convection/mixing process. We consider simulation #1 (see Table 1) as the case.

In the initial phase (t = 30 s, #1 in Fig. 2), Rayleigh-Taylor instabilities at magma interface develop into buoyant plumes. By increasing the viscosity, interface destabilization is delayed. A similar behaviour is observed by decreasing volatiles.

Theoretical analysis^39^ provides an approximated expression for the characteristic plume wavelength, already employed in problems related to magma convection dynamics^10^:

1 $$\begin{array}{cc}\lambda =4\pi {(\frac{{\nu }^{2}}{gA})}^{\mathrm{1/3}} & A=\frac{{\rho }_{dacite}-{\rho }_{andesite}}{{\rho }_{dacite}+{\rho }_{andesite}}\end{array}$$

where *ν* is the kinematic viscosity of the magma at the interface, *A* is the Atwood number. In^40^, the author provides the following theoretical expression for the wavelength when the viscosity of the fluid in the upper layer is larger than that of the lower melt layer, and the lower melt layer is generated by melting upon heating across a pre-existing melt-solid interface.

2 $$\lambda =2.289\,\pi \,{\mu }_{r}^{\mathrm{2/3}}\sqrt{\frac{v{\mu }_{l}}{g\,{\rm{\Delta }}\rho }}$$

where *μ*_*r*_ is the ratio between the larger and smaller melt viscosities, *μ*_*l*_ is the viscosity of the lower (less viscous) melt, Δ*ρ* is the numerator of the Atwood number and v is the velocity of the advance of the melt layer upon melting of the underlying solid.

By employing quantities computed from our initial conditions, the computed plume wavelengths by equation (1) correspond to expected number of plumes in the range 2–10, decreasing with increasing viscosity. That compares well with the simulated dynamics in Fig. 2, where from 3 to 9 plumes (decreasing with increasing viscosity) are initially formed at magma interface. Such plumes can then further interact with each other as well as with the overall fluid flow, either merging or splitting. Although the experimental set up and the problem studied in^40^ is different from the set up in our numerical simulations, we find that a velocity in the range 4–6 m/s, comparable to the average velocities from our numerical simulations, provides a perfect matching between the number of plumes predicted from equation (2), and those observed in the simulations.

**Figure 3:**
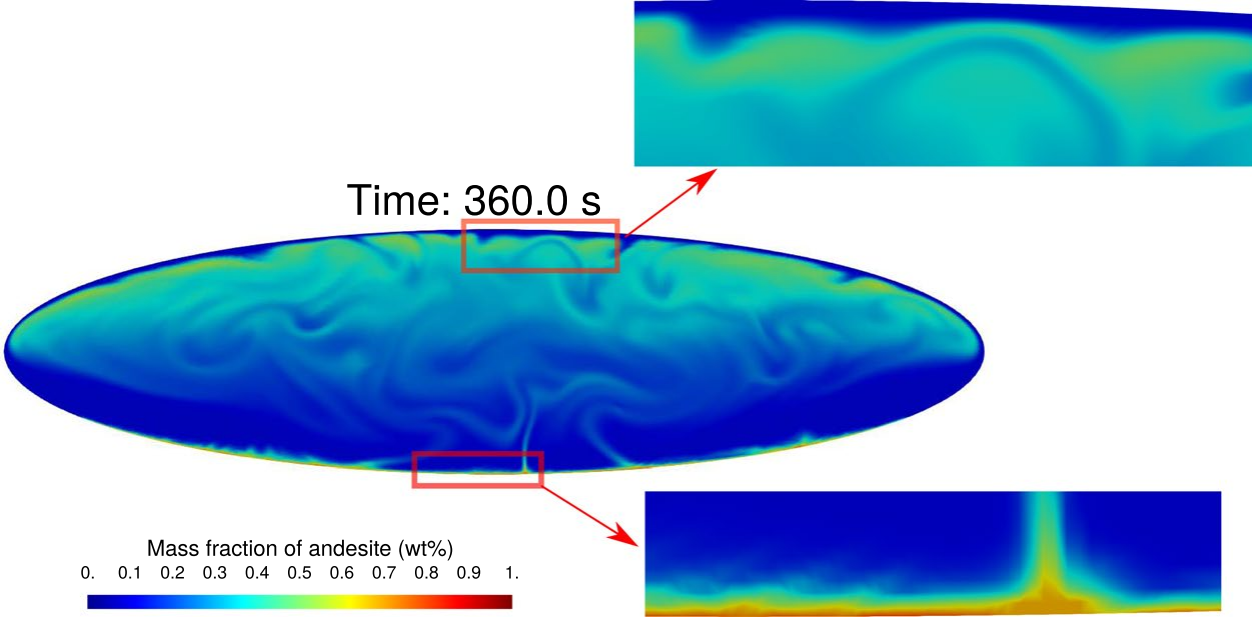
Distribution of andesitic composition during second phase at time t = 360 s for simulation #1. The zoomed views show the boundary layers of andesite at the bottom and dacite at the top of the chamber.

In the second phase, the hotter and less dense andesitic magma penetrates through the dacitic magma generating plume structures (*e*.*g*. see t = 70 s in the reference simulation #1 in Fig. 2). The plumes rise towards the chamber roof producing a structure approximately symmetric with respect to the vertical axes, with the largest plume at the centre of the interface (primary plume), where viscous forces are the least. The rise of the plumes is delayed by increasing viscosity or decreasing volatiles. Viscosity and volatiles have a strong effect on the wave number of the destabilizing interface, hence on the number of plumes and the thickness of the neck of the plumes. In the reference case #1 it is possible to identify 11 uprising plumes, while they reduce to 5 by increasing viscosity (#2, #3) or decreasing volatiles (#4). The thickness of the neck of the primary plume varies from 3 to 9 m, increasing with viscosity and decreasing with amount of volatiles. Furthermore, while in the increased viscosity (#2, #3) and volatile-poor (#4) cases the plumes of the andesitic magma go straight to the top of the chamber, weakly interacting with the dacitic magma, in case #1 and #5 the plumes merge with each other, interact with the surrounding magma and rise in a zigzag pattern resulting in overall increased chaoticity and efficiency of mixing. In the volatile rich-case (#5), although the rising plumes expand more and show a faster dynamics with respect to the reference simulation #1, their qualitative behaviour looks quite similar. By the end of the second phase the system has overturned, i.e., andesite has reached the upper part of the chamber and the colder and denser dacitic magma has been displaced to chamber bottom. We observe that portions of andesite and dacite magmas stick along the walls forming boundary layers (Fig. 3).

**Figure 4:**
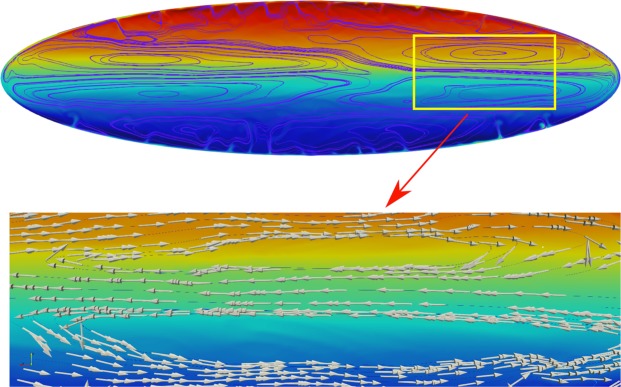
The convective rolls represented by velocity streamlines and the orientation of velocity vector for two consecutive, counter-rotating cells in simulation #1 at time t = 6310 s.

**Figure 5:**
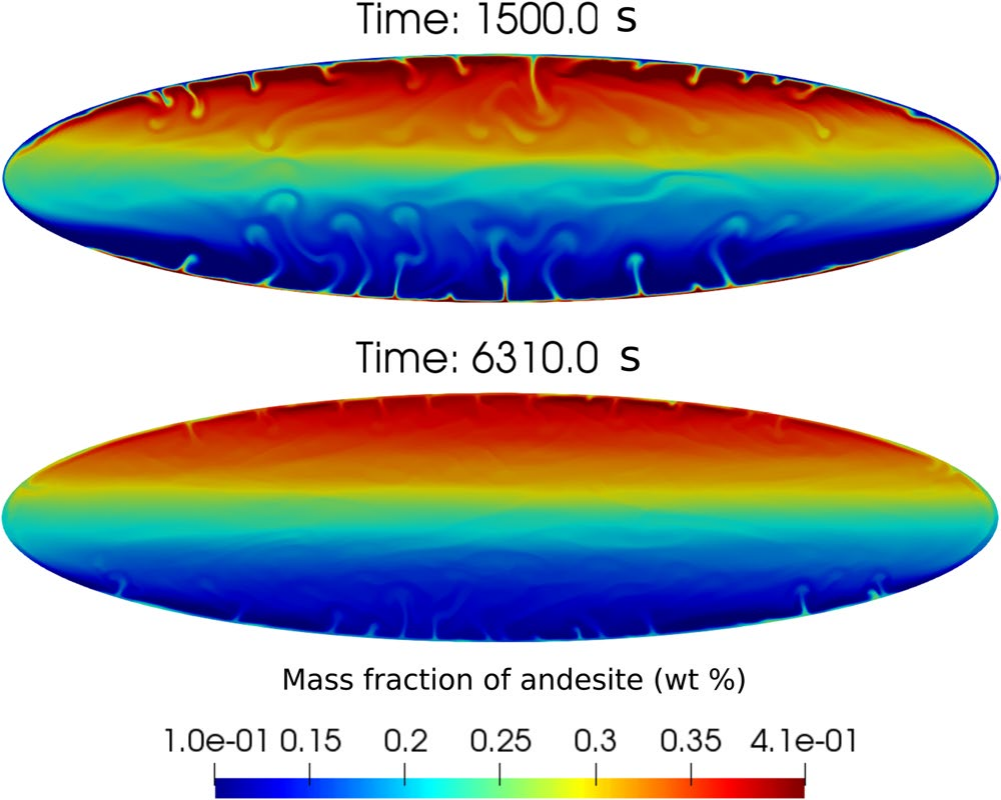
Temporal evolution of plume generation during third phase by andesite at the bottom boundary layer and dacite at the top boundary layer for simulation #1. The boundary layers become thinner with time.

By the third phase, the system has evolved into a stratified chamber with the upper and lower portions dominated by the light andesite and dense dacite magma, respectively. Such two portions tend to remain separated, due to the development of separate convective cells recirculating magma within each chamber portion (Fig. 4). Circulation velocities rapidly decrease to very low values of order 10^−3^ m/s. As a consequence, magma mixing in the third phase is largely hindered and poorly effective, both within each recirculating region and across them, where it is limited by the long time scales of chemical diffusion. The two boundary layers at chamber top and bottom (Fig. 3) correspond to the areas with largest density contrasts during this phase. Such density contrasts are gravitationally unstable, and generate sinking/rising plumes of close-to-pure dacite and andesite that penetrate the convective regions enhancing local mixing, without crossing the boundary between upper/lower recirculating portions (Fig. 5). This process results in thinning of the boundary layers from their initial thickness of order 1–10 m, larger for more viscous, more volatile-depleted magmas. The movies of the simulations, figures with snapshots of pressure distribution at different times for cases #1,3,5, and the power spectrum of composition related to three phases of cases #1,4 are provided as Supplementary Material. In particular, because the overall mass in the chamber is constant, and the vertical extent of the simulated domain is relatively small, pressure changes are minor during the simulated dynamics (Figs S4–S6 in supplementary material). Previous work analysed magma chamber - dyke systems extending vertically for several kms, highlighting heterogeneously distributed, significant pressure changes^16^.

## Discussion

### Magma mixing and compositional evolution in magma chambers

Magma convection and mixing resulting from the unstable configuration in Fig. 1 develops through complex dynamics in which we identify three main phases characterized by (i) interface destabilization and plume onset, (ii) plume rise and efficient convection/mixing, and (iii) magma stratification and development of separate, horizontally elongated convective cells. Such a three-phase evolutionary dynamics is maintained when considering ample variations in magma volatile contents and overall magma viscosity increase by one order of magnitude. However, such changes in the conditions and properties of magmas involved in the convection processes bring about substantial differences in terms of mixing efficiency and time-scales, that are analysed and discussed below.

It is worth reminding here that our definition of magma mixing relates to the spatial resolution of the numerical simulations, which is of order 1 m. Therefore, having an element with a certain relative proportion of the two magma types does not tell us much about the extent of chemical vs. mechanical mixing (the latter often called “magma mingling”). Computed element Peclet numbers, representing the ratio of advective vs. diffusive mass transfer, are of order 10^9^ during phase 2, decreasing to 10^7^ during phase 3, showing that advection largely dominates over diffusion, therefore, that mechanical dominates over chemical magma mixing during our simulated times. In summary, the mixing time scales that we show in this work only refer to mechanical mingling over a spatial scale of order 1 m, and should not be confused with time scales for chemical mixing occurring at the scale of molecules, that we do not take into account and are expected to be much longer.

Before discussing further our numerical results, it is useful to put them into more context in light of the assumptions and limitations in our work. Two aspects in particular are worth of being considered: (1) the neglect of crystals, and (2) the 2D assumption.

We show in the Methods section that within the range of simulated conditions, gas bubbles are not expected to deform efficiently, therefore, they are not expected to introduce any non-Newtonian effect (they still increase the Newtonian viscosity, what we account for as described in the Methods). Neglecting crystals, therefore, has the consequence that no complex rheology is needed. However, a number of real magmas carry significant amounts of crystals, and more importantly, crystal can be heterogeneously distributed in a magma chamber^41^, giving origin to regions with complex mechanical behaviour. If similar regions, like for example mushy regions close to chamber walls, exist inside magma chambers, they are expected to result in more complex circulation patterns, not accounted for in this work.

Similarly, a 3D geometry would affect the circulation patterns, e.g., in terms of vorticity^42^, duration of each phase identified during convection and mixing, etc. While accounting for such complexities requires a substantial step forward in computation capacity, the present results are expected to capture the essence of the overall dynamics, and be qualitatively similar to the 3D case.

Figure 2 shows that depending on the specific conditions and properties characterizing the two magma types involved in convection and mixing, the volatile-rich, light andesitic magma initially placed at chamber bottom may be largely lost, or instead mostly preserved, as an individual end-member component during the long-lasting phase three. Lower volatile contents of magmas, and larger overall magma viscosity, tend to favor preservation of andesite-rich magma at chamber top (e.g., cases #3 and #4 in Fig. 2). By referring to previous mixing experiments where buoyant plumes formed as a consequence of fluid injection into a tank^43^ rather than from Rayleigh-Taylor instability over a gravitationally unstable interface as in the present simulations, we have computed a mixing efficiency, representing the degree (in the 0–1 range) of mixing between the different fluids under convection. We find that the mixing efficiency is about 0.5 in simulation #1, decreasing to about 0.1 in simulation #4. Such a decrease in mixing efficiency is paralleled by a decrease of Re (computed as in^20^) from 10^3^ to 10.

**Figure 6:**
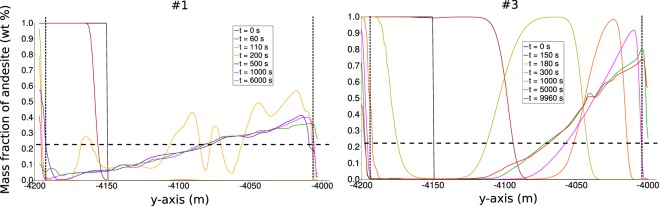
Compositional profile along x = −100 m for simulation cases #1 and #3. The portions outside the vertical dashed black lines are boundary layers. The hybrid composition is displayed by the horizontal dashed line.

To better illustrate the dynamics of mixing and the effects of the specific characteristics of the involved magmas, Fig. 6 shows the vertical distribution of composition along a profile 100 m to the left with respect to mid-chamber axis, at many different times, for the two cases #1 (intermediate volatile content) and #3 (same as #1 with 10-fold increased viscosity). The profiles for cases #2, #4 and #5 are provided as Supplementary Material. Except for the boundary layers and the regions in their immediate proximity, that continue to evolve over the entire simulation times, in both cases a time is reached, corresponding to onset of phase 3, when no significant compositional evolution is further observed (cfr. lines corresponding to time 500 s or larger for case #1, and 5000 s or larger for case #3). The onset of phase 3 is substantially delayed, from less than 10 minutes to more than 1h20’, due to increased magma viscosity. During the slowly evolving phase 3, the low-viscosity case #1 shows that the chamber bottom is occupied by > 90 wt% dacitic magma, progressively evolving to a 60–70 wt% dacitic magma at chamber top. By contrast, the high-viscosity case #3 shows an ample bottom region, up to nearly half chamber height, occupied by nearly pure dacite, which progresses into a 70–80 wt% andesitic magma at chamber top. We conclude that magma mixing during phase 2 has been much more effective for the low viscosity case #1, whereas for the high viscosity case #3 it has left sharp compositional diversities that are maintained during the long-lasting phase 3. Figure 2 shows that decreasing the volatile content in the two magmas (case #4) has an overall effect similar to that of increasing viscosity as from case #1 to case #3.

The circulation patterns characterizing phase 3 and illustrated in Fig. 4 correspond to velocities of order 10^−3^ m/s and *Re* ≪ 1 (creeping flow), therefore, they are poorly effective in locally homogenizing the magmatic composition. Only minor plumes generated from the boundary layers (Figs 3 and 5) contribute to increasing further local compositional heterogeneities during phase 3. Boundary layers form close to chamber walls due to local high shear stress, largely delaying local magma displacement by buoyancy forces, giving rise to locally large compositional gradients progressively adsorbed during phase 3. The effect of boundary layers, however, is limited to a region close to the chamber walls (Fig. 6) and progressively wanes during phase 3 (Fig. 5).

**Figure 7:**
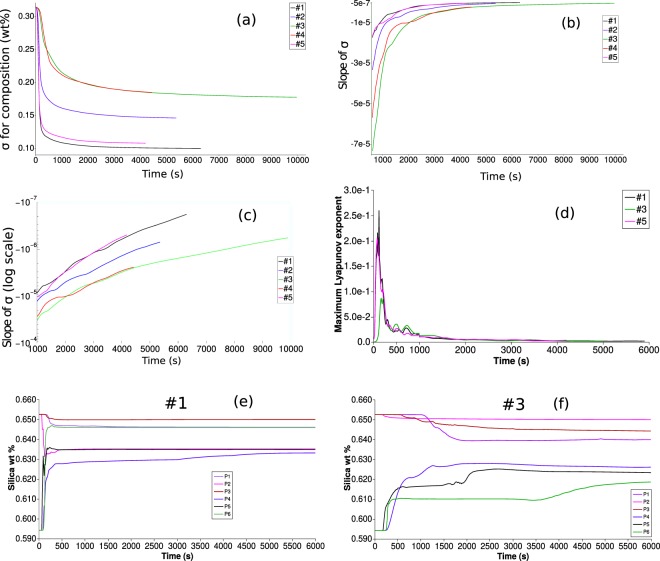
(**a**) Temporal evolution of standard deviation (*σ*) of composition (andesite wt%). (**b**) Temporal evolution of slopes of *σ*. (**c**) Semi-log plot of rate of *σ*. (**d**) Time series for maximum finite time Lyapunov exponent (FTLE). (**e**) Time series for silica (wt%) at Lagrangian tracer points (P1–P6 displayed in Fig. 1) for case #1. (**f**) Time series for silica (wt%) at Lagrangian tracer points for case #3.

Figure 7a shows the evolution of overall compositional heterogeneities (at the resolution scale of the numerical simulations), in terms of the quantity ***σ*** representing the standard deviation of the distribution of the wt% of andesite within about 150,000 elements constituting the computational domain in the magma chamber. Similar technique based on the decay of concentration variance has been used in the experiments^26^. The initial rapid decay represents efficient mixing dynamics during phase 2 (the initial delay close to time zero represents phase 1). The onset of phase 3 corresponds to flattening in the trend of sigma, which is delayed and much less abrupt for the low volatiles, high viscosity cases #3 and #4. Figure 7b shows the evolution of the slope of ***σ***, or the rate of compositional homogenization in the magma chamber, across phase 2 and during phase 3. After an initial rapid rate increase (or decrease of its absolute value), further increase is progressively slower. Figure 7c shows that the rates tend to an exponential behaviour during phase 3 (corresponding to a straight line on the semi-log plot in the figure). Therefore, the absolute value of the rate of compositional homogenization progressively decreases, implying that the curves in Fig. 7a progressively flatten with time. In other words, the compositional heterogeneities existing in the magma chamber at onset of phase 3 do not evolve with time, rather, they are destined to be maintained as a stable dynamic configuration. Similar conclusions are reached by referring to local scale evolution through computation of Lyupanov exponents, instead of referring to global evolution as from the overall compositional spectrum in the chamber. In particular, we refer to the maximum value of finite-time Lyupanov Exponents^44^ (FTLE):

3 $$FTLE=\frac{1}{T}\,\mathrm{log}\,\frac{dz(t+T)}{dz(t)}$$

where *dz*(*t*) and *dz*(*t* + *T*) are distances between Lagrangian trajectories at time *t* and *t* + *T*. Figure 7d shows the computed maximum FTLE values, while Fig. 7e,f show examples of Lagrangian trajectories corresponding to points P1–6 in Fig. 1 (further discussed below). As for the global analysis from the evolution of the compositional spectrum at Fig. 7a–d shows that an initial phase of chaotic mixing (high FTLE values) is rapidly followed by a much longer phase with maximum FTLE values approaching zero, meaning that local stretching is quickly hindered everywhere in the chamber, and that magma mixing is no longer effective during the dynamically stable phase 3. Such a stability can be altered only by external factors, such as magma degassing (causing local density increase), new ingression of volatile rich magma into the chamber (causing new destabilization and the repetition of the convection and mixing patterns described here), or by magma crystallization and associated processes, e.g., concentration of volatiles with respect to the melt and further degassing, and migration of crystals or crystal-rich mush. These processes are expected to occur, although they are likely to be effective on a time scale longer than that of our simulations and establishment of a long-lived, dynamically stable phase 3.

### Crystal textures

Although we neglect crystals in the present simulations, calculation of the Lagrangian paths at Fig. 7e,f allows us to speculate on the textural evolution of crystals that may be transported with individual melt parcels. This is more justified the more crystals are poorly abundant, thus not introducing substantial differences in the calculated flow patterns (e.g., from^45^, non-Newtonian effects are poor or negligible for crystal volume fractions less than 10%), and as long as the crystals are sufficiently small to guarantee effective mechanical coupling with the melt.

Repeated crystal resorption and growth are ubiquitous occurrences during magmatic processes^10,46^, reflecting rate-limited melt-solid reactions towards new thermodynamic equilibrium as the local P-T-composition conditions evolve. The Damkolher number^10^, representing the ratio of advection to growth or dissolution timescales, allows an evaluation of the local dominant processes affecting crystal textures:

4 $$\begin{array}{cc}D{a}_{g}=\frac{{t}_{adv}}{{t}_{g}}; & D{a}_{d}=\frac{{t}_{adv}}{{t}_{d}}\end{array}$$

where *t*_*g*_ and *t*_*d*_ are the timescale of growth and dissolution estimated, respectively, over a typical length scale of 10 and 1 micron (reasonable for microprobe analyses). For plagioclase crystals in andesitic-dacitic melts, *t*_*g*_ is estimated in the range 10^2^–10^3^ s^47^ and *t*_*d*_ is of the order of 10 s^48^. Timescales of advection can be estimated from the Lagrangian paths in Fig. 7e,f, being 100 s during the rapid initial compositional changes in simulation #1 (Fig. 7e), up to 1000–2000 s for the initial transient phase in simulation #3 (Fig. 7f). In the former case, *Da*_*g*_ < 1 while *Da*_*d*_ can vary from 1 to 10, suggesting that under such conditions, dissolution dominates. After the initial dissolution-dominated phase, the conditions are stabilized again as the individual magma parcels are trapped within vortexes and further magma mixing is hindered. During that phase, *Da*_*g*_ easily exceeds 1, and crystal growth dominates. Therefore, the conditions of simulation #1 suggest plagioclase textures showing dissolution of a core, followed by stable growth of euhedral rims, either more An-rich or less An-rich than the core itself, depending on if the original plagioclase grew in the andesitic or dacitic magma. Similar textures characterized by resorption followed by euhedral growth are common; e.g., they are observed at Andagua monogenetic centre^46,49^, where they are interpreted as the result of a single magma recharge event. In the case of simulation #3, the initial, one order of magnitude longer transient phase results in *Da*_*g*_ and *Da*_*d*_ numbers that both largely span the range from <1 to ≫1. Therefore, more complex textural patterns are expected, with growth and resorption acting together and possibly resulting in multiple resorption surfaces and zoning patterns, before stable conditions are reached and euhedral crystal growth takes place. Subsequent transient phases, e.g. visible in the trends of particle P6 at around 4000 s, can result in further dissolution-growth before a new phase of euhedral growth is established, giving origin to a compositionally different plagioclase rim. Similar complex patterns of crystal resorption and growth are also common, e.g., they are observed in feldspar crystals in andesitic samples from El Misti volcano^46^, where they are interpreted as multiple recharging events. Our results cannot rule out that interpretation, but show that both simple patterns of dissolution followed by growth, and more complex patterns of growth and dissolution, can be associated with single overturning in a magma chamber due to individual magma recharge events. FTLE calculations shown in Fig. 7d allow to estimate the maximum dispersal of two initially adjacent magma parcels, which is of order 10 m for simulation #1, and 100 m for simulations #3,5. Therefore, crystals initially in equilibrium with either andesite or dacite can be found close to each other, and display a range of textural evolutions depending on their initial composition and individual paths during one single event of magma convection and mixing, like the ones simulated here. Similar features are described in the andesites from Mount Hood^49^. Textural and geochemical analyses show strong evidences for two distinct populations of plagioclase that are intimately intermingled, one formed in equilibrium with relatively mafic melts and a second one presenting disequilibrium textures characterized by patterns of dissolution and growth^49^. This is consistent with a stratified magma chamber characterized by the coexistence of magmas from nearly pure to variably mixed end-member compositions.

### Implications for magmatic reconstructions and volcanic hazard

Convection and mixing following a gravitationally unstable interface between a volatile-rich, buoyant andesitic magma underlying a partially degassed dacitic magma, are found to result in the establishment of variable degrees of dynamically stable, long-lived compositional heterogeneities. Volatile poor magmas, or more viscous magmas, favour larger degrees of heterogeneity and preserve individual end-member components, although the buoyant (less abundant in our simulations) and more primitive andesitic magma is never maintained as an individual component at the spatial scale of our numerical resolution.

The above conclusions have relevant consequences for the interpretation of geochemical and petrologic data from real eruptions. Besides the expanded opportunities for interpreting complex crystal textures as discussed above, they bring substantial relevance in the reconstructed or forecasted scenarios for hazard purposes. A first direct implication of the present results is that the common observation of coexisting magmas with different degree of chemical differentiation in the products of individual volcanic eruptions, does not necessarily reflect a triggering mechanism due to new arrival of magma in a shallow chamber. In fact, compositionally different magmas can coexist for long time in a shallow chamber during the dynamically stable phase 3. An order of magnitude estimate of the time required for chemical homogenization from the long-lasting conditions during phase 3 depends on the spatial scale of compositional heterogeneities expected to characterize the magmatic mixture. For the more viscous, less volatile rich cases #3 and #4, the dynamics of convection result in substantial decrease of velocities and much less efficient mechanical mixing, revealed by halving the number of rising plumes, triplicating the average width of individual plume neck, and largely reducing the vorticity and chaotic nature of convection and mixing (Fig. 2), finally leading to increased preservation of compositional end-members. It is reasonable to expect that such conditions also lead to increased spatial scale of compositional heterogeneities. With en-mass andesite-dacite diffusion coefficient of order 10^−9^ m^2^/s, a simple order-of-magnitude evaluation of Fick’s law suggests that chemical (diffusive) mixing can efficiently homogenize the magmatic mixture over a time-scale of order hours to days if the spatial scale of heterogeneities is of order 1 cm or less. That time-scale is increased to order of months to years if the spatial scale of heterogeneities is of order 10 cm or more. Over the spatial scale of magma chamber heterogeneities described for case #3 in Fig. 6, magma homogenization by diffusion is not expected to be efficient within a time comparable to that of major changes in the magmatic system. Our results provide therefore a common framework for reconciling largely variable degrees of mixing^3,5,6,50^, and mixing time scales from hours to years, found by different authors^26–28^ for different magmatic systems.

Long-lived compositional heterogeneities in magma chambers, including the existence of regions hosting pure or nearly pure end-member components, may profoundly affect the reconstructions of processes preceding or triggering a volcanic eruption, and the volcanic hazard implications thereby. Time scales obtained from chemical disequilibrium between crystals and melts, as well as from chemical diffusion profiles, are often ascribed to the time spent from new magma arrival into a shallow chamber to eruption^26^. Notably, such time scales turn out to be of order tens of minutes to tens of hours in a number of cases^11,26,29^. In terms of volcanic hazards, such short times imply that the time for volcanic emergency operations, e.g., for city evacuation, since when we may detect signs of new magma arrival at shallow level to a possible consequential eruption, can be largely insufficient; e.g., the foreseen time for the evacuation of the heavily urbanized areas subject to volcanic risk from a Campi Flegrei eruption, close to Naples, is three days (DPC evacuation plan). Those results invariably assume that mixing between compositionally different magmas is a consequence of new magma arrivals from depth. However, our numerical simulations reveal that largely different magmas can coexist in a shallow chamber, and that such coexistence can be maintained for long. It is natural to expect that further magma migration from a shallow chamber towards the surface, and rapid withdrawal of magma during an eruption, largely destroy the dynamically stable configuration of phase 3, repeatedly shuffling the magma over the hours and days of the eruption. As a consequence, portions of magmas that were initially distant inside the chamber would repeatedly be brought into contact, and chemical disequilibria over short time scales comparable to that of the eruption may be effective during the eruption itself.

It is necessary to clarify here that we do not neglect the possibility of new magma arrivals shortly preceding an eruption. The point we stress, and that derives from our simulation results, is that evidence of magma mixing over short time scales does not necessarily reflect such new arrivals, because different magmas can coexist for long time inside a magma chamber; and that magma mixing over short time scales of order tens of minutes to tens of hours can be a consequence, and not necessarily a trigger, of a volcanic eruption. In terms of volcanic hazards and emergency preparedness, short mixing times would signify extremely reduced or no time for safety operations if they relate to new magma arrivals from depth and consequent eruption occurrence; while that relation does not hold, and the time for operations is not necessarily strict, if short mixing times reflect instead long-lived compositional heterogeneities inside the chamber, shuffled and mixed further over the time scale of the eruption itself. Finally, a corollary to our results is that highly heterogeneous conditions in a magma chamber prior to an eruption make point data from selected samples of the erupted magma poorly informative on the pre-eruptive conditions. Rather, sampling and chemical analyses should be treated in the frame of statistical analysis, which may require new approaches to define the statistical representativeness of samples and the related sampling techniques.

## Methods

We model the magma as a mixture of two components, each one characterised by a melt with given composition, and a given amount of volatiles. Volatile species are constituted by H_2_O and CO_2_, which are assumed in thermodynamic equilibrium with the melt. Melt-volatile multicomponent thermodynamics are computed through the non-ideal, compositionally-dependent model in^51^, which allows computation of the dissolved and exsolved volatile amounts, and of the composition of the gas phase, as a function of pressure, temperature, melt composition and total amount of each volatile species.

The FEM numerical model solves the compressible flow of the magma mixture resulting from interaction between the individual magma components. The equations governing mass conservation for each component, and momentum and energy balance for the magma mixture, are:

5 $$\begin{array}{cc}Mass & \frac{\partial (\rho Y)}{\partial t}+\nabla \cdot (\rho Y\otimes v)=\nabla \cdot J,\end{array}$$

6 $$\begin{array}{cc}Momentum & \frac{\partial (\rho v)}{\partial t}+\nabla \cdot (\rho v\otimes v)=\nabla \cdot \sigma +\rho g,\end{array}$$

7 $$\begin{array}{cc}Energy & \frac{\partial (\rho E)}{\partial t}+\nabla \cdot (\rho Ev)=\nabla \cdot (\sigma v-q-(h^{\prime} J)^{\prime} )+\rho (g\cdot v),\end{array}$$

where *Y* = {*y*_1_,*y*_2_}′ is the vector of mass fractions with *y*_2_ = 1−*y*_1_; *v* = {*v*_1_,*v*_2_}′ is the velocity vector; *ρ* is mixture density; *g* is gravitational acceleration; *E* = *c*_*v*_ * *T* + |*v*|/2 is total specific energy, with *c*_*v*_ being the specific heat at constant volume; T is temperature; *h* is the vector of specific enthalpies for the components.

Mass diffusion flux is modelled with Fick’s law as *J* = −*ρD*∇ · *Y*, where *D* is the mass diffusion coefficient matrix. Viscous flux is modelled as $\sigma =\mu (\nabla v+{(\nabla v)}^{\text{'}})-\frac{2}{3}\mu (\nabla \cdot v)I-pI$, where *p* is pressure and *μ* is the viscosity of the mixture. The heat flux is modelled by Fourier’s law: *q* = −*κ*∇*T*, where *κ* is thermal conductivity. Phase separation is not accounted for in the above transport equations. Post-processing of the numerical results show that Stokes numbers for crystals (up to cm size) and gas bubbles (for the computed gas volume fractions, and with assumed bubble number density as low as 10^14^ m^−3^) are very low, of order 10^−4^ or less, meaning that mechanical phase separation is negligible under the conditions of our simulations. Local clustering of gas bubbles (or crystals, not included in the simulations) and gas bubble coalescence, which may increase buoyancy and favour phase separation, constitute further complexities not accounted for in the modelling.

The physical properties of magma are modelled as a function of local pressure, temperature and composition in the space-time domain. Each magma component is modelled as a mixture of silicate melt and volatiles (H_2_O + CO_2_). The density of the volatile free silicate melt is modelled according to^52^ and the density of the dissolved water is computed by the model of^53^. For the gas phase the ideal gas equation was used. The magmatic mixture density is computed as ${\rho }^{-1}={\sum }_{i}{y}_{i}/{\rho }_{i}$, where *y*_*i*_ and *ρ*_*i*_ are the mass fraction and density of the *i*th component, respectively. The viscosity of each magma component is modelled as in^54^, with the effect of non-deformable gas bubbles accounted for by the model of^55^.

The model accounts for non-Newtonian rheology as due to deformable gas bubbles and presence of crystals. In the present simulations, gas bubble deformation is ruled out by computed Capillary numbers (*Ca* = *μv*/*σ*) much less than 1 at any point in time and space. That fact, together with melt-dominated conditions examined in this work, allow neglecting non-Newtonian behaviours, resulting in increased speed and efficiency of the calculations. Comparing with the results in^45^, Newtonian behaviour is approximated for crystal contents less than 10–20 vol%, to which, therefore, our numerical simulations refer. The viscosity of the two-component mixture is modelled as $\mu ={\sum }_{i}{x}_{i}{\mu }_{i}$, where *x*_*i*_ and *μ*_*i*_ are, respectively, the mole fraction and the viscosity of the *i* th mixture component. The specific heat at constant pressure for the melt is computed as ${c}_{p}={\sum }_{j}{x}_{j}{c}_{pj}$, where *c*_*pj*_ and *x*_*j*_ are, respectively, the specific heat at constant pressure and mole fraction for the *j* th oxide subcomponent (Table 1^56^). The specific heat at constant volume, *c*_*v*_ is computed as *c*_*v*_ = *c*_*p*_ − *α*^2^*T*/(*βρ*). In this work we use a constant thermal conductivity^56^, *κ* = 1.2 *Wm*^−1^*K*^−1^. Variables and parameters used in this work are listed in Table 2.

**Table 2:** Variables and parameters used in the simulations.

*E*	Total Specific Energy	[*Jkg*−1]	
*J*	diffusion flux	[*kgm*^−2^*s*^−1^]	
*T*	temperature	[*K*]	
*Y*	mass fraction		
*h*	specific enthalpies	[*Jkg*^−1^]	
*p*	pressure	[*Pa*]	
*q*	heat flux	[*Wm*^−2^]	
*t*	time	[*s*]	
*v*	velocity	[*ms*^−1^]	
*x*	mole fraction		
*μ*	viscosity	[*Pas*]	
*ρ*	density	[*kgm*^−3^]	
*σ*	stress tensor	[*Nm*^−2^]	
*c*_*p*_	specific heat at constant pressure	[*Jkg*^−1^*K*−1]	
*c*_*v*_	specific heat at constant volume	[*Jkg*^−1^*K*−1]	
*D*	mass diffusion coefficient	[*m*^2^*s*^−1^]	10^−9^
*g*	gravitational acceleration	[*m*/*s*^2^]	9.81
*κ*	thermal conductivity	[*Wm*^−1^*K*^−1^]	1.2

### Equation of state for magma mixture

The magma mixture density is computed as Amagat-Leduc law of additive volumes and can be written as a function of mass fraction of two end member magmas:

8 $$\frac{1}{\rho }=\frac{{y}_{1}}{{\rho }_{1}}+\frac{{y}_{2}}{{\rho }_{2}}$$

where *y*_1_, *ρ*_1_ and *y*_2_, *ρ*_2_ are respectively the mass fractions in the mixture and densities of andesite and dacite magmas. Each magma in turn is composed of subcomponents: silica melt and volatiles (*H*_2_*O* + *CO*_2_). The density of the andesite magma can be computed as

9 $$\frac{1}{{\rho }_{1}}=\frac{{\hat{y}}_{an{d}_{-}melt}}{{\rho }_{an{d}_{-}melt}(p,T)}+\frac{{\hat{y}}_{{H}_{2}O(l)}}{{\rho }_{{H}_{2}O(l)}(p,T)}+\frac{{\hat{y}}_{C{O}_{2}(l)}}{{\rho }_{C{O}_{2}(l)}(p,T)}+\frac{T}{p}({\hat{y}}_{{H}_{2}O(g)}{R}_{{H}_{2}O}+{\hat{y}}_{C{O}_{2}(g)}{R}_{C{O}_{2}})$$

where ${\hat{y}}_{i}$ is the mass fraction of the *i* th subcomponent of andesite magma and *R*_*i*_ is the specific gas constant. If *y*_*i*_ is the mass fraction of the *i* th subcomponent in the mixture (andesite + dacite) then ${y}_{i}={\hat{y}}_{i}{y}_{1}$. Substituting ${\hat{y}}_{i}={y}_{i}/{y}_{1}$ in the above equation we get

10 $$\frac{{y}_{1}}{{\rho }_{1}}=\frac{{y}_{an{d}_{-}melt}}{{\rho }_{an{d}_{-}melt}(p,T)}+\frac{{y}_{{H}_{2}O(l)}}{{\rho }_{{H}_{2}O(l)}(p,T)}+\frac{{y}_{C{O}_{2}(l)}}{{\rho }_{C{O}_{2}(l)}(p,T)}+\frac{T}{p}({y}_{{H}_{2}O(g)}{R}_{{H}_{2}O}+{y}_{C{O}_{2}(g)}{R}_{C{O}_{2}})$$

Similarly, following eqs (9–10) for dacite magma we get

11 $$\frac{{y}_{2}}{{\rho }_{2}}=\frac{{y}_{dac\_melt}}{{\rho }_{dac\_melt}(p,T)}+\frac{{y}_{{H}_{2}O(l)}}{{\rho }_{{H}_{2}O(l)}(p,T)}+\frac{{y}_{C{O}_{2}(l)}}{{\rho }_{C{O}_{2}(l)}(p,T)}+\frac{T}{p}({y}_{{H}_{2}O(g)}{R}_{{H}_{2}O}+{y}_{C{O}_{2}(g)}{R}_{C{O}_{2}})$$

substituting equations (10) and (11) in equation (8) the equation of state for the magma mixture is given as

$$\begin{array}{rcl}\frac{1}{\rho } & = & \frac{{y}_{an{d}_{-}melt}}{{\rho }_{an{d}_{-}melt}(p,T)}+\frac{{y}_{da{c}_{-}melt}}{{\rho }_{da{c}_{-}melt}(p,T)}\\  &  & +\,2(\frac{{y}_{{H}_{2}O(l)}}{{\rho }_{{H}_{2}O(l)}(p,T)}+\frac{{y}_{C{O}_{2}(l)}}{{\rho }_{C{O}_{2}(l)}(p,T)}\\  &  & +\,\frac{T}{p}({R}_{{H}_{2}O}{y}_{{H}_{2}O(g)}+{R}_{C{O}_{2}}{y}_{C{O}_{2}(g)}))\end{array}$$

### Numerical scheme

An about uniform mesh composed of ∼150,000 elements with an average edge length h ∼1 m is selected after performing a grid convergence study. In all simulations, the starting time step is set to 0.01 s and gradually increased approaching the steady state. The simulations are run with in-house software GALES, already used for simulating magma mixing in volcanic environments^12,16,30^. The software is written in object-oriented C++ and parallelized using Open-MPI. A large part of the code uses the libraries of Trilinos package which provides a variety of routines for matrices, vectors, finite element assembly process and the linear solvers^57^. The numerical scheme is based on stabilized space-time finite element method which is well established in engineering literature for both compressible and incompressible flows. The solution scheme is implicit and eqs (5–7) are solved in a monolithic way for transformed set of variables {*p*,*v*,*T*,*Y*}. Validation of the software has been done on several flow problems elsewhere^30,31,58,59^, spanning conditions from 1 to 2D, one-component to multi-component, incompressible to compressible flow, and large Re range.

## Supplementary information

Supplementary movie of case1

Supplementary movie of case2

Supplementary movie of case3

Supplementary movie of case4

Supplementary movie of case5

Supplementary movie of case1 with lagrangian tracers

Supplementary movie of case3 with lagrangian tracers

Supplementary file

## Data Availability

The movies of numerical simulations are available as Supplementary Material. All data generated or analysed during this study is available from the corresponding authors upon request.
