# Supplementary material for: Long-lived compositional heterogeneities in magma chambers, and implications for volcanic hazard
Source: Sci Rep. 2019 Mar 1;9:3321. doi: 10.1038/s41598-019-40160-1 (PMC6397211; doi:10.1038/s41598-019-40160-1)
Supplement: Supplementary file 8 — Supplementary file [file 41598_2019_40160_MOESM8_ESM.pdf]

Supplementary material

Long-lived compositional heterogeneities in magma chambers, and implications for volcanic hazard

Deepak Garg[1]*, Paolo Papale[1], Simone Colucci[1], and Antonella Longo[1]

[1]Istituto Nazionale di Geofisica e Vulcanologia, Sezione di Pisa, Via della Faggiola 32, 56126 Pisa, Italy

*Correspondence: deepak.garg@ingv.it

Contents

Supplementary Figures

- **Figure S1**
- **Figure S2**
- **Figure S3**
- **Figure S4**
- **Figure S5**
- **Figure S6**
- **Figure S7**
- **Figure S8**
- **Figure S9**
- **Figure S10**

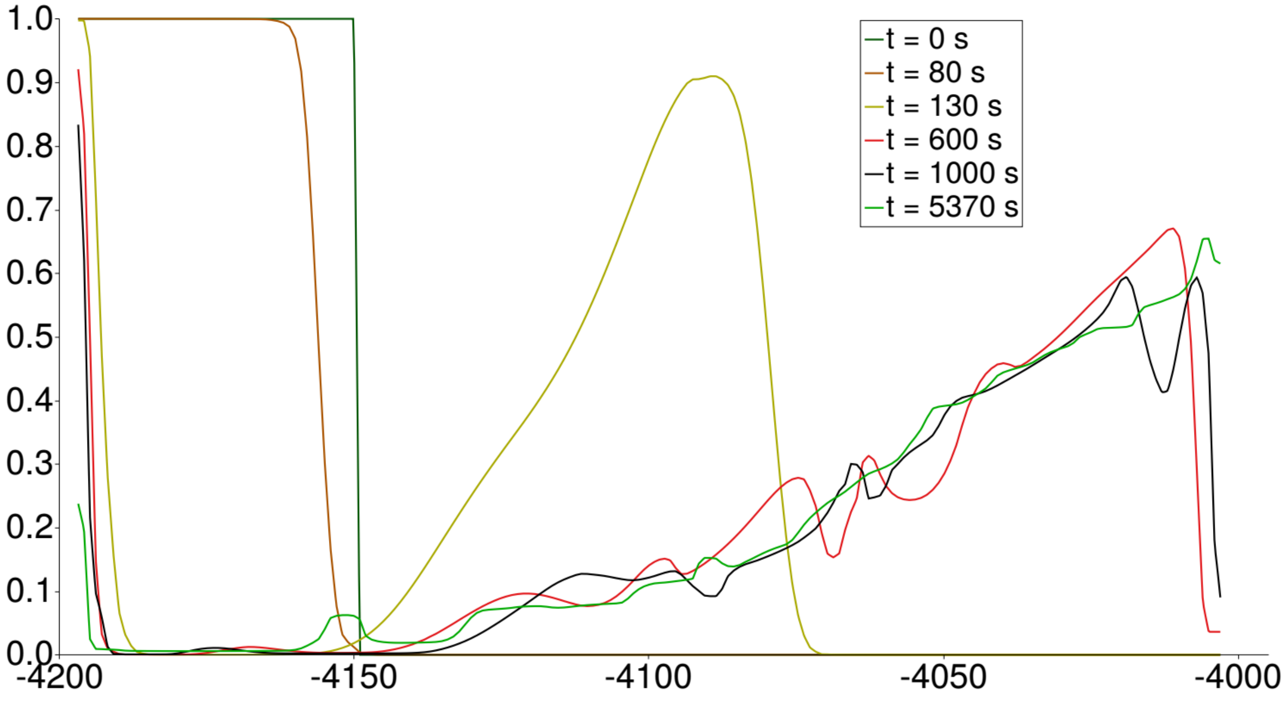

Figure S1: Compositional profile along x = -100 m for simulation case #2. The portions outside the vertical dashed black lines are boundary layers. The hybrid composition is displayed by the horizontal dashed line.

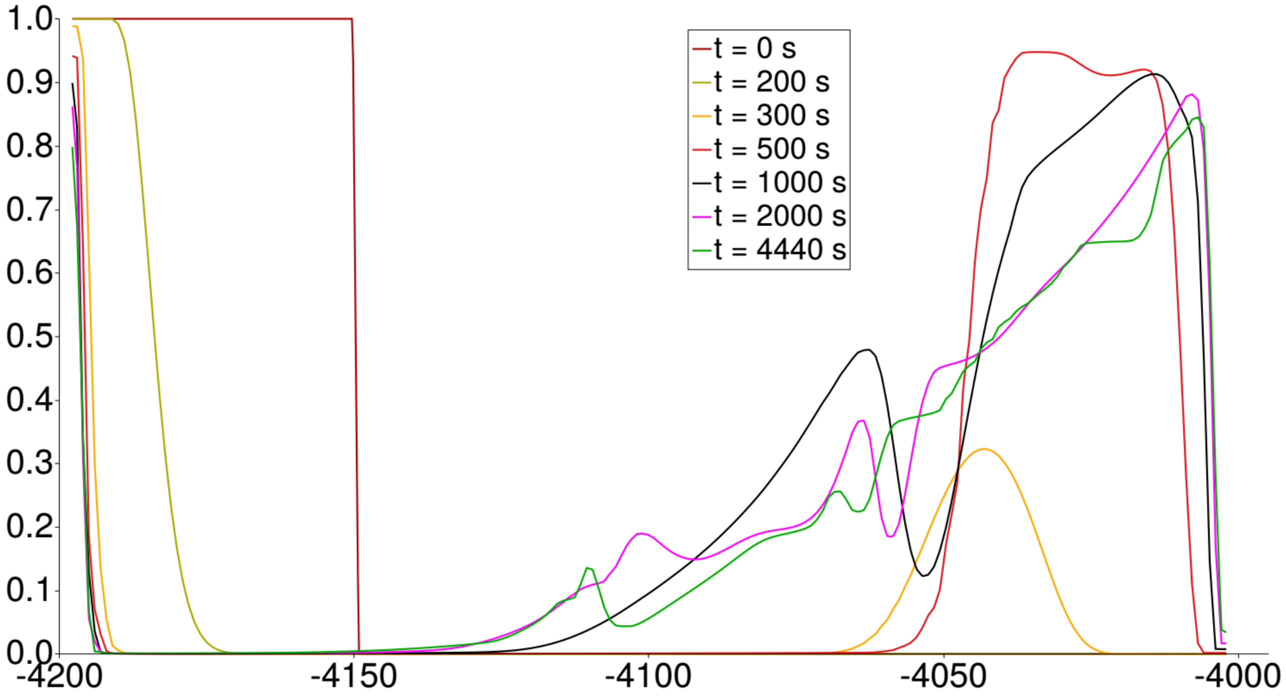

Figure S2: Compositional profile along x = -100 m for simulation case #4. The portions outside the vertical dashed black lines are boundary layers. The hybrid composition is displayed by the horizontal dashed line.

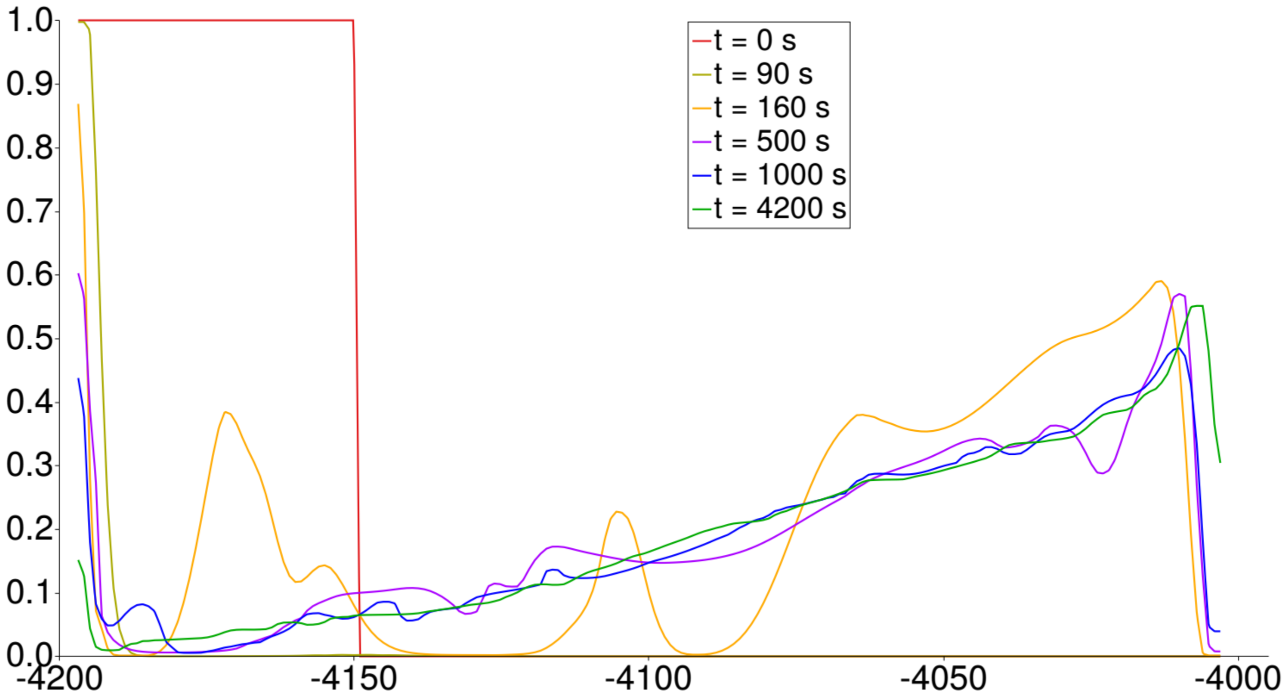

Figure S3: Compositional profile along x = -100 m for simulation case #5. The portions outside the vertical dashed black lines are boundary layers. The hybrid composition is displayed by the horizontal dashed line.

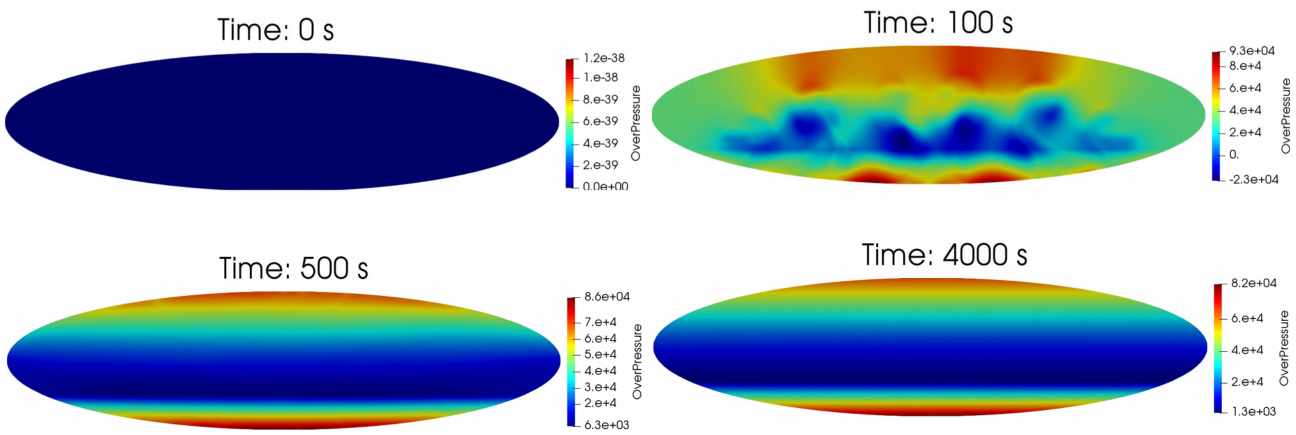

Figure S4: Distribution of overpressure at different times for case #1

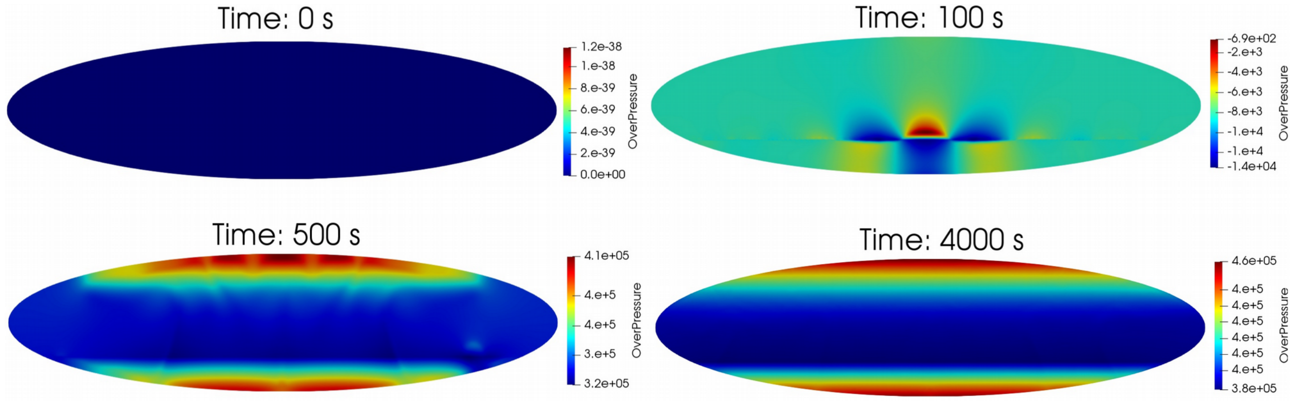

Figure S5: Distribution of overpressure at different times for case #3

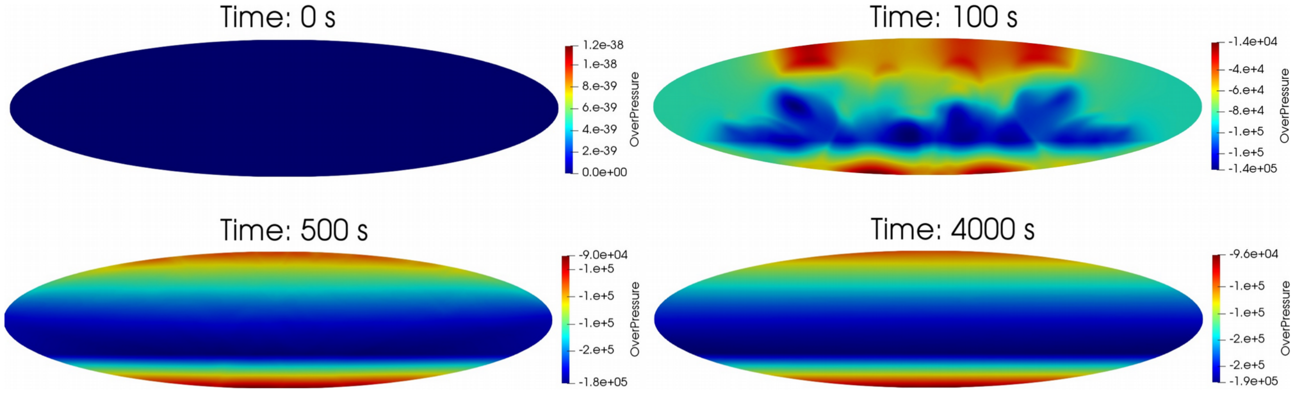

Figure S6: Distribution of overpressure at different times for case #5

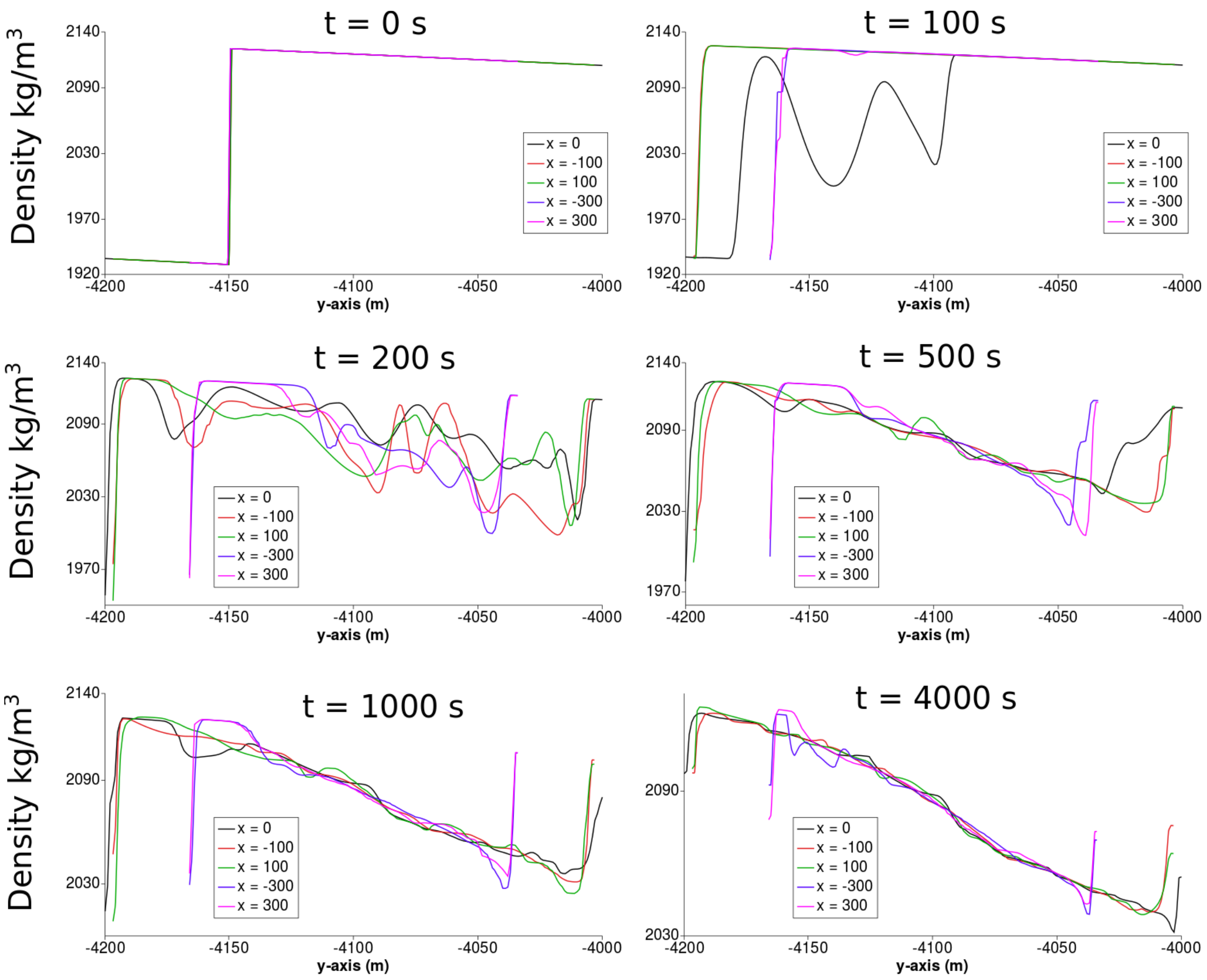

Figure S7: Density profiles along lines x = 0, -100, 100, -300, 300 for simulation #1

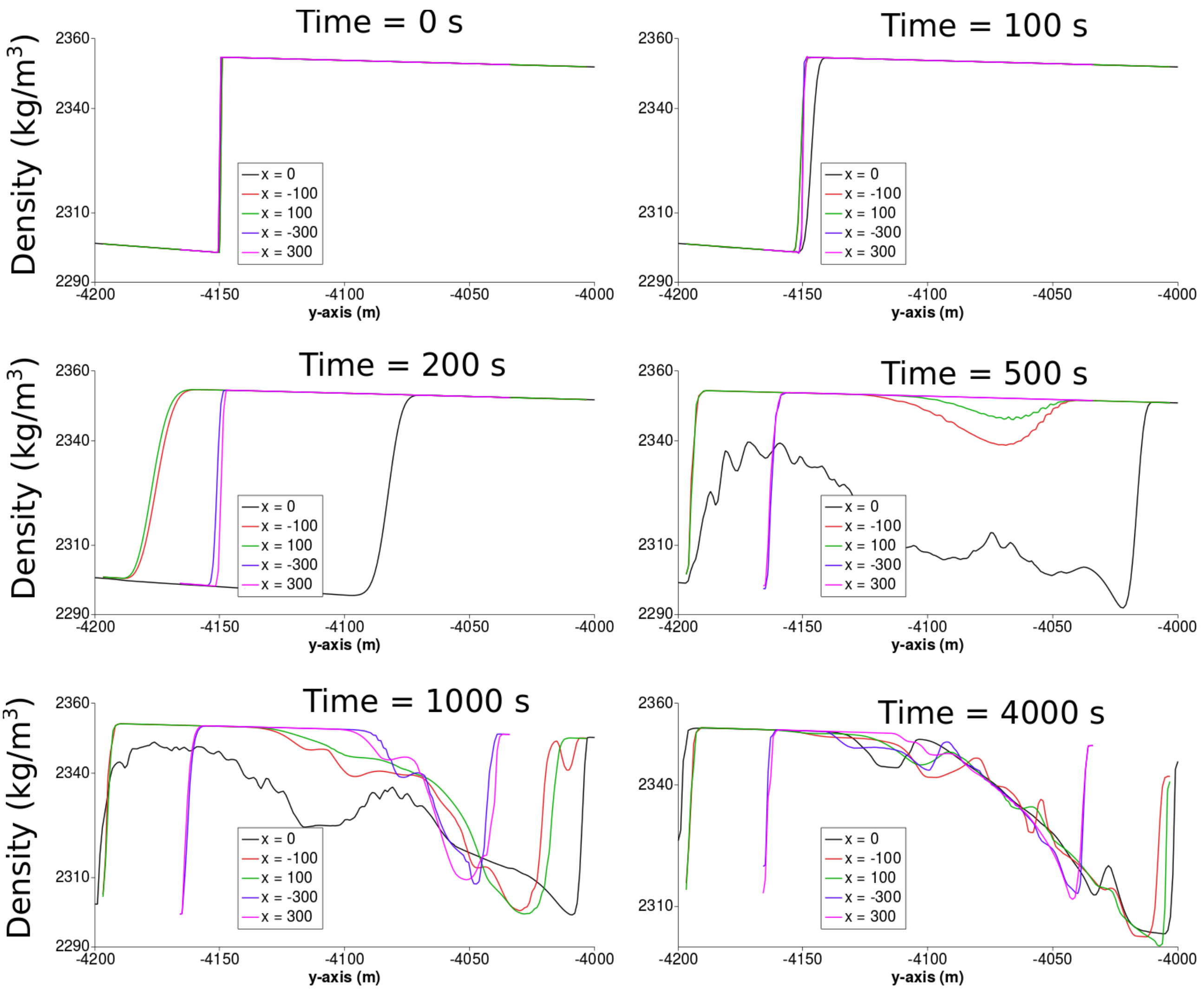

Figure S8: Density profiles along lines x = 0, -100, 100, -300, 300 for simulation #4

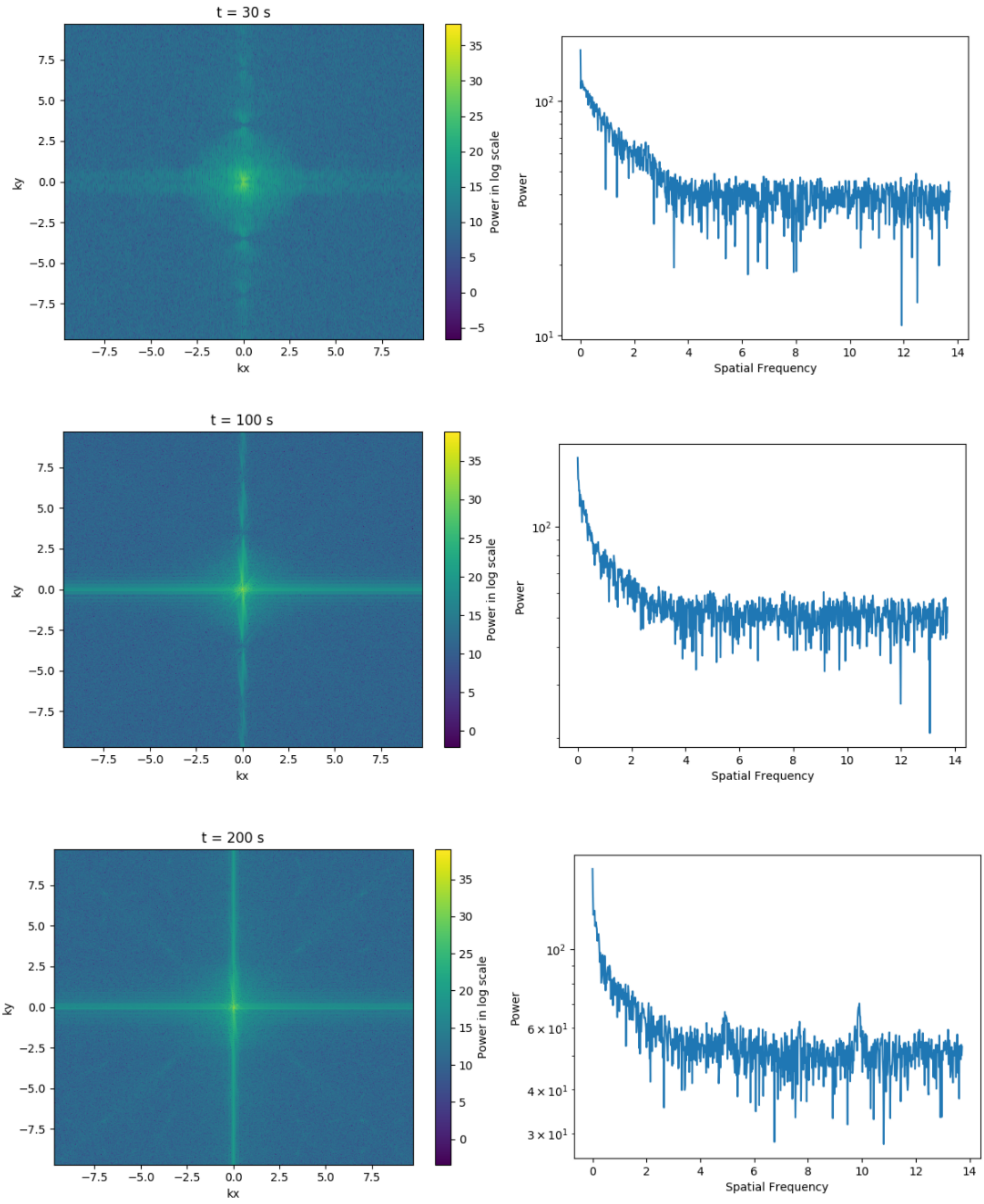

Figure S9: Power spectrum of compositional field for simulation #1. Left panel shows 2D PSD at different times. Right panel shows 1D PSD.

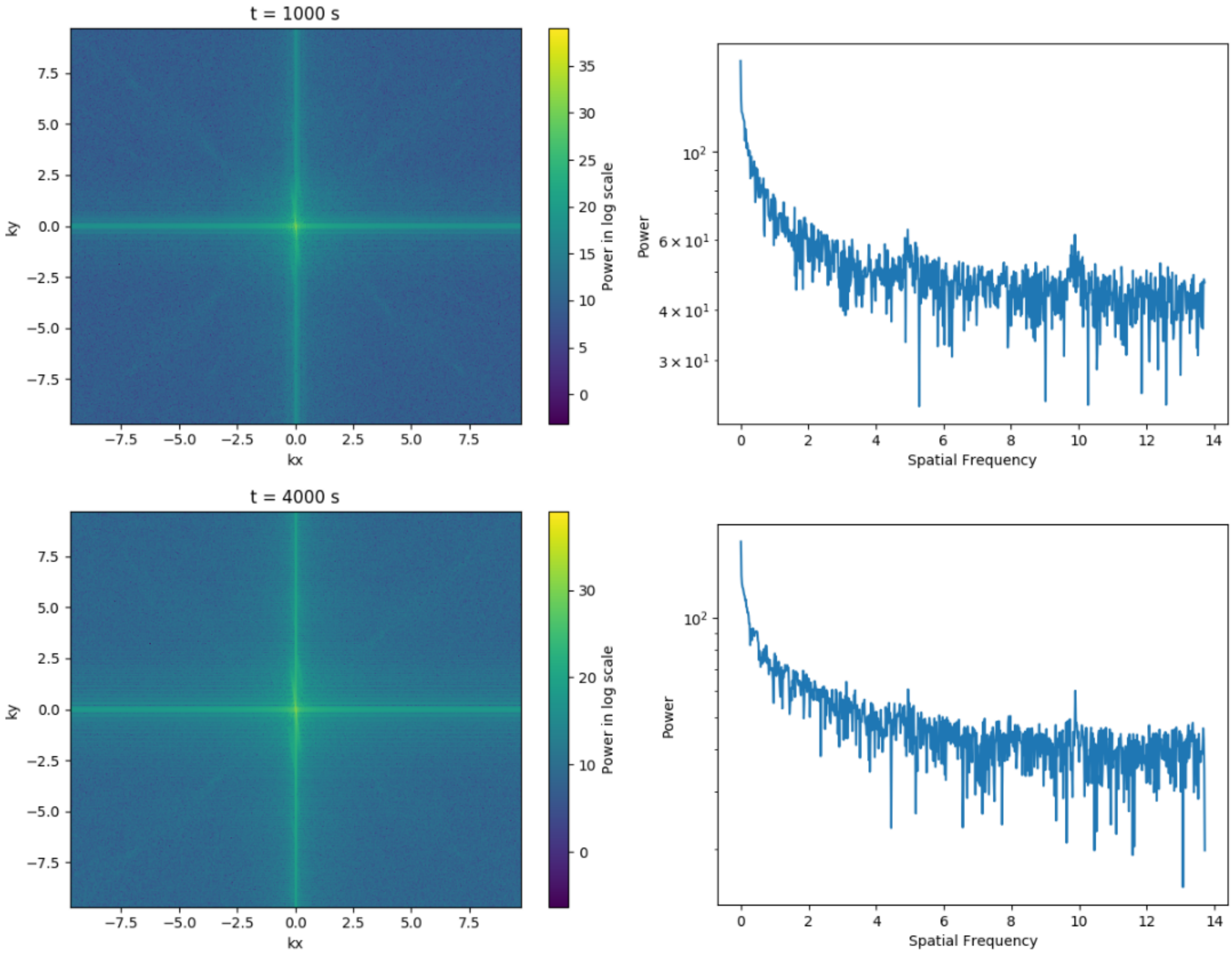

Figure S9: Continued

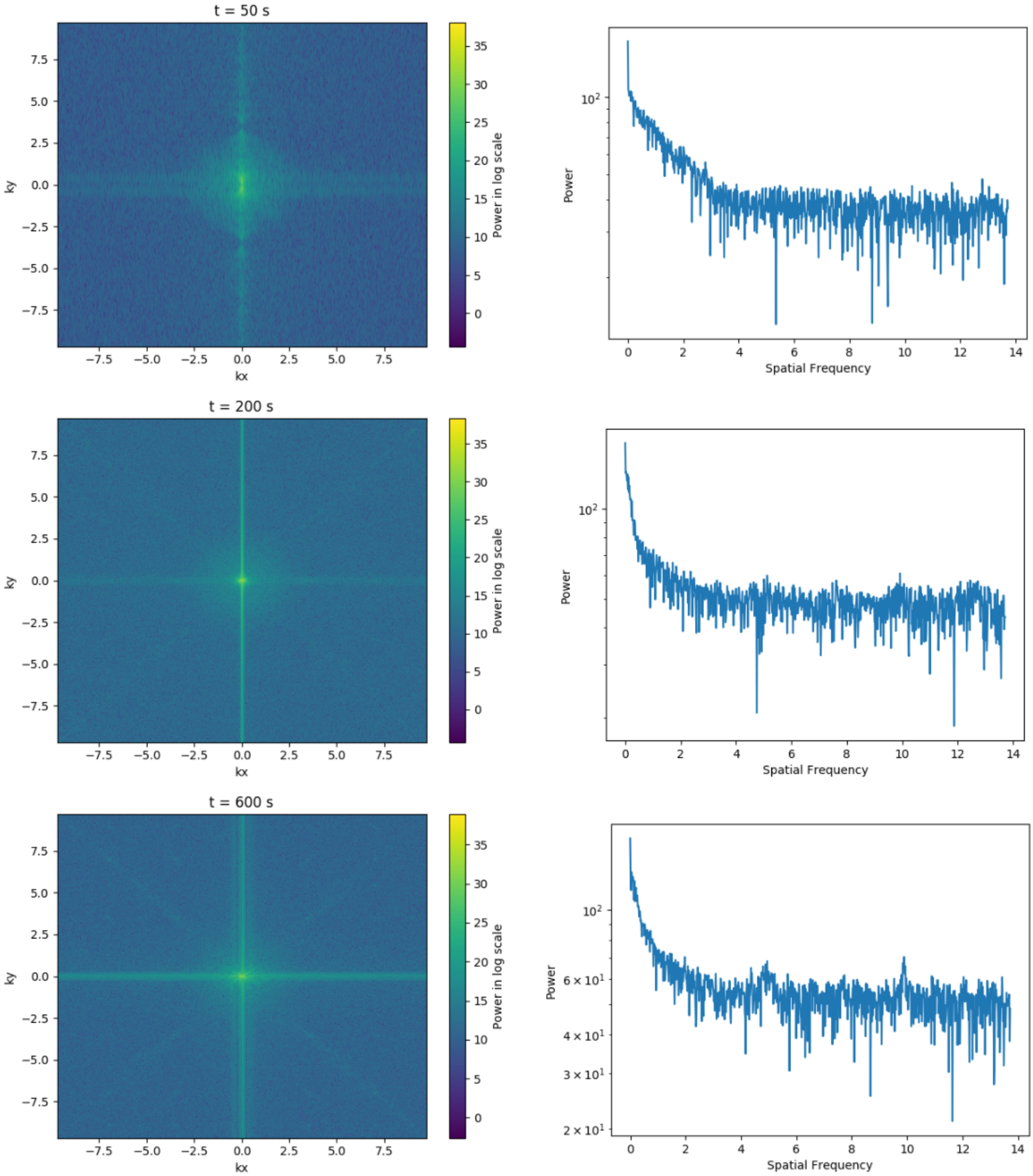

Figure S10: Power spectrum of compositional field for simulation #4. Left panel shows 2D PSD at different times. Right panel shows 1D PSD.

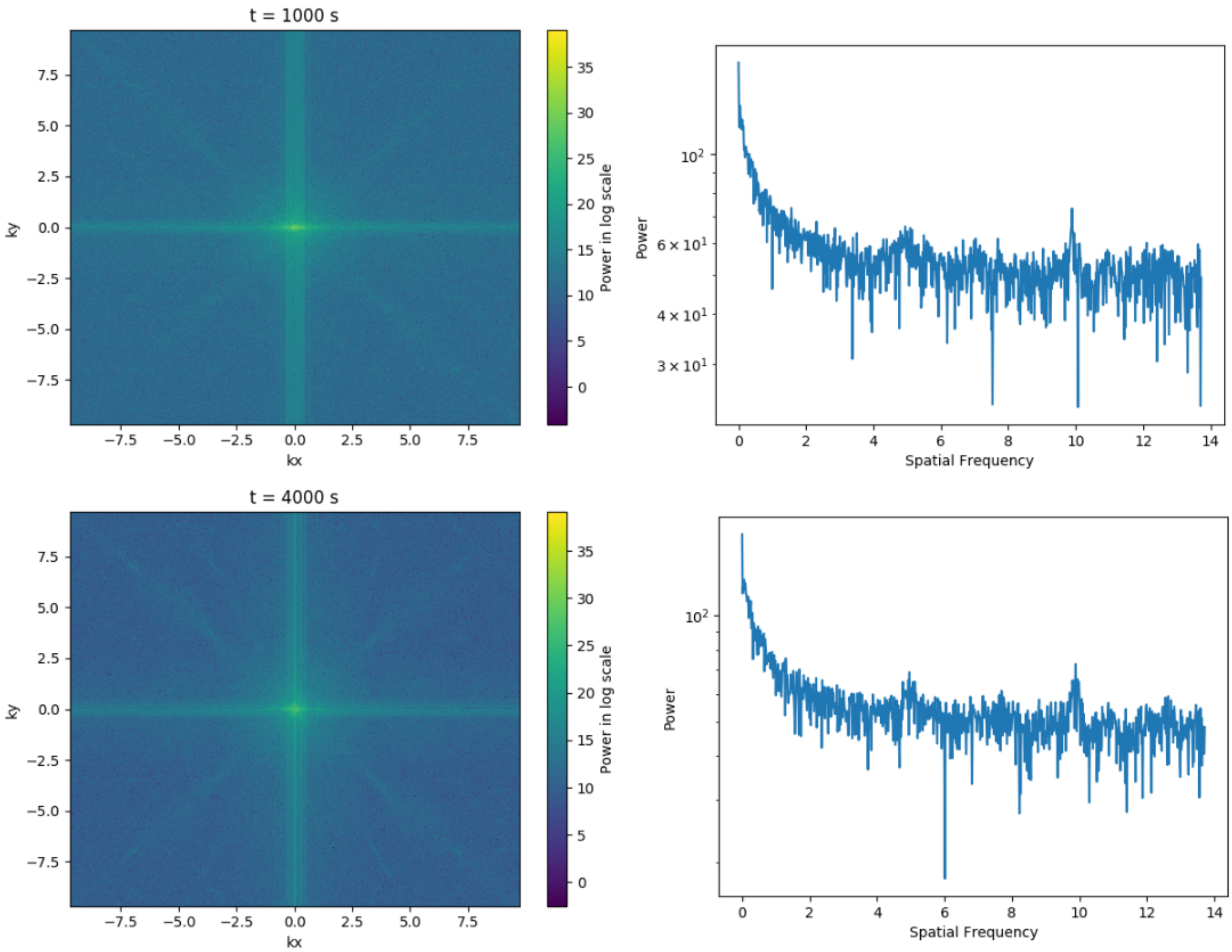

Figure S10: Continued

Method for computation of power spectrum:

The power spectrum of composition field is obtained by applying 2D Fast Fourier Transformation (FFT2) on the images of 175 m^2 square domain generated at different times. We chose five images of composition representing all three phases. We use fft2 and fftshift inbuilt functions of python. The number of pixels per unit meter and the frequency domain in x and y directions are computed based on the image resolution. The size of pixel is 0.103 m^2. 1D PSD is computed by taking the magnitude of powers in x and y directions.
